# Ageing European lobsters (*Homarus gammarus*) using DNA methylation of evolutionarily conserved ribosomal DNA

**DOI:** 10.1111/eva.13296

**Published:** 2021-09-23

**Authors:** Eleanor A. Fairfield, David S. Richardson, Carly L. Daniels, Christopher L. Butler, Ewen Bell, Martin I. Taylor

**Affiliations:** ^1^ School of Biological Sciences University of East Anglia Norwich UK; ^2^ The National Lobster Hatchery Padstow UK; ^3^ The Centre for Environment, Fisheries and Aquaculture Science Lowestoft UK

**Keywords:** chronological age, DNA methylation, epigenetic clock, fisheries, lobster, ribosomal DNA

## Abstract

Crustaceans are notoriously difficult to age because of their indeterminate growth and the moulting of their exoskeleton throughout life. The poor knowledge of population age structure in crustaceans therefore hampers accurate assessment of population dynamics and consequently sustainable fisheries management. Quantification of DNA methylation of the evolutionarily conserved ribosomal DNA (rDNA) may allow for age prediction across diverse species. However, the rDNA epigenetic clock remains to be tested in crustaceans, despite its potential to inform both ecological and evolutionary understanding, as well as conservation and management practices. Here, patterns of rDNA methylation with age were measured across 5154 bp of rDNA corresponding to 355 quality‐filtered loci in the economically important European lobster (*Homarus gammarus*). Across 0‐ to 51‐month‐old lobsters (*n* = 155), there was a significant linear relationship between age and percentage rDNA methylation in claw tissue at 60% of quality‐filtered loci (*n* = 214). An Elastic Net regression model using 46 loci allowed for the accurate and precise age estimation of individuals (*R* ^2^ = 0.98; standard deviation = 1.6 months). Applying this ageing model to antennal DNA from wild lobsters of unknown age (*n* = 38) resulted in predicted ages that are concordant with estimates of minimum size at age in the wild (mean estimated age = 40.1 months; range 32.8–55.7 months). Overall, the rDNA epigenetic clock shows potential as a novel, nonlethal ageing technique for European lobsters. However, further validation is required across a wider range of known‐age individuals and tissue types before the model can be used in fisheries management.

## 1 INTRODUCTION

Knowledge of the age structure of animal populations is fundamental to understanding their ecology, evolution and conservation (De Paoli‐Iseppi et al., 2017; Jarman et al., 2015). Animal age can be used to predict mortality risk, reproductive potential and susceptibility to parasites (De Paoli‐Iseppi et al., 2019). In fisheries management, age structure is a key predictor of population dynamics and is therefore crucial for their sustainable management (Campana, 2001). However, animal age is often very difficult to measure. Some animals exhibit physical features that are correlated with age, such as growth rings in fish otoliths (Panella, 1971) and bivalve shells (Kilada et al., 2009), and tooth length in deer (Pérez‐Barbería et al., 2014). However, many animals lack such characteristics and accurate age estimates are often only attainable through expensive tracking or marking studies (De Paoli‐Iseppi et al., 2019). Unfortunately, these approaches are not practical for many species, especially those that are long‐lived or inhabit environments that are difficult to access.

Recently, molecular markers of age have generated interest among those looking to develop affordable, accurate, nonlethal and minimally invasive methods for estimating animal age (De Paoli‐Iseppi et al., 2017; Jarman et al., 2015). These involve measuring a feature of an individual's DNA or RNA, or associated molecules, that changes consistently over time. Telomere length, which declines throughout life in many species (reviewed by Dunshea et al., 2011), was the first genetic marker of age to receive widespread attention among molecular ecologists. However, despite its initial promise, telomere length does not accurately predict chronological age in many animals, likely because of the complex interplay of genetic and environmental effects (Barrett et al., 2013; Bize et al., 2009; Monaghan & Haussmann, 2006). Other molecular methods of age determination have been suggested, particularly those based on changes to DNA damage or abundance throughout life (e.g. mitochondrial DNA heteroplasmy or copy number). However, to date, none of these methods have successfully been applied as molecular markers of age in a wild animal (Jarman et al., 2015; Jebb et al., 2018).

A promising and more recently explored avenue for developing a molecular ageing assay is DNA methylation, whereby methyl groups are added to DNA, almost exclusively where cytosine precedes guanine (CpGs; Jones et al., 2015). This epigenetic change plays an important role in controlling gene expression (Schübeler, 2015). A gradual decline in genome‐wide (global) methylation with increasing age has been observed in many taxa, including fish (*Oncorhynchus gorbuscha*: Berdyshev et al., 1967), mammals (*Homo sapiens*: Fuke et al., 2004; *Mus musculus*: Wilson et al., 1987), invertebrates (*Chlamys farreri*: Lian et al., 2015) and birds (*Gallus gallus*: Gryzinska et al., 2013). This age‐related decline in global DNA methylation, combined with increasing variance among individuals, is known as ‘epigenetic drift’ (Field et al., 2018). At individual CpGs, the amount of methylation can undergo a positive (hypermethylation) or negative (hypomethylation) linear relationship with chronological age in humans and other animals (reviewed by De Paoli‐Iseppi et al., 2017). Such relationships may remain linear irrespective of how long an animal lives; that is, the ‘tick rate’ of CpG methylation differs according to lifespan (Field et al., 2018; Lowe et al., 2018). Site‐specific CpG predictors of age (epigenetic clocks), often based on a small subset of age‐informative CpGs, are highly correlated with age and display low margins of error in every species studied to date, including a number of different bat species (Wilkinson et al., 2021; Wright et al., 2018), short‐tailed shearwaters (*Ardenna tenuirostris*: De Paoli‐Iseppi et al., 2019), chimpanzees (*Pan troglodytes*: Ito et al., 2018; Guevara et al., 2020), bottlenose dolphins (*Tursiops truncatus*: Beal et al., 2019), European bass (*Dicentrarchus labrax*: Anastasiadi & Piferrer, 2020), humans (Bocklandt et al., 2011; Horvath, 2013), mice (Han et al., 2018) and humpback whales (*Megaptera novaeangliae*: Polanowski et al., 2014). Being able to estimate chronological age by measuring CpG methylation using a handful of loci has made it possible to implement epigenetic ageing tools in various areas of applied research, including human forensics (Shabani et al., 2018) and marine science (Beal et al., 2019; Polanowski et al., 2014).

Until recently, a significant barrier to developing an ageing tool in nonmodel organisms based on epigenetic clocks was the need to have high‐quality, full‐length genomic data for either the species of interest or at least a closely related species. There are next‐generation sequencing (NGS) approaches that make it possible to attain site‐level methylation data across entire genomes without prior genomic information, but these methods are costly (Kurdyukov & Bullock, 2016). Developing a more cost‐effective, targeted ageing assay based on a limited number of CpGs relies on being able to identify differentially methylated sites using existing genetic data (De Paoli‐Iseppi et al., 2019). Extensive gene‐level information on age‐related DNA methylation exists for humans (Horvath, 2013), so many studies on the existence of epigenetic clocks in nonmodel animals have targeted orthologous sequences of the age‐related genes identified in humans (Beal et al., 2019; Polanowski et al., 2014). However, this approach is only feasible for closely related species, which likely explains the bias towards mammals in epigenetic clock studies. This necessity has been potentially circumvented by the discovery of an epigenetic clock that may be applicable across the animal kingdom (Wang & Lemos, 2019). This new ageing tool is based on methylated cytosines in ribosomal DNA (rDNA), which is the most evolutionarily conserved region of the genome (Wang & Lemos, 2019). Across ca. 13,000 bp of rDNA sequence in mice, 620 age‐informative CpGs (66.8%) were discovered. Many CpGs were found to occur in distantly related taxa; for example, more than 70% of human CpGs in the 18S and 5.8S genes of rDNA are found in species as divergent as zebrafish (*Danio rerio*).

Crustacean fisheries are a major industry with substantial benefits for human livelihoods and food security worldwide. Crustacean catch has the highest export value per live weight of any aquatic animal group, with a 22% global share by trade value (Food & Agriculture Organization [FAO], 2020). However, concerns have been raised over the long‐term sustainability of crustacean fisheries. The assessment and regulation of crustacean stocks is challenging because it is currently impossible to accurately estimate crustacean age and therefore make reliable predictions about population dynamics (Anderson et al., 2011; Boudreau & Worm, 2012). Additional stock assessment uncertainty arises for crustaceans caught in traps because catches may be size biased and not representative of the population. Crustaceans are difficult to age because they moult throughout their lives and show indeterminate growth but with extensive individual variation in size at age (Kilada & Driscoll, 2017; Vogt, 2012). Several alternative methods for estimating crustacean age have been investigated (reviewed by Kilada & Driscoll, 2017; Vogt, 2012), but none have been adopted for routine use due to technical limitations (tag and recapture, lipofuscin content—the accumulation of a pigment associated with cellular degradation and ageing in most eukaryotes), limited or no association with chronological age (lipofuscin content, telomere length), the need for lethal sampling (lipofuscin content and growth bands), or because questions remain as to whether individuals are affected by moulting (growth bands; Becker et al., 2018; Huntsberger et al., 2020). Therefore, a reliable and accurate ageing method is urgently needed for crustacean fisheries management and would have considerable positive economic and conservation impacts. In a recent review of future genetic tools for lobster management, DNA methylation‐based markers were highlighted as a possible solution to age estimation (Silva et al., 2019).

The European lobster (*Homarus gammarus*) is an economically important species harvested across its range in the shallow, coastal areas of the northeast Atlantic Ocean. Lobster landings are valued at more than £44 million per annum to the UK alone (Marine Management Organisation [MMO], 2019). Stock assessments are currently based on tracking the change in length frequencies across years at the population level to estimate future resilience to fishing. The European lobster has an estimated lifespan of 42–72 years (Sheehy et al., 1999), and length is not an accurate predictor of age (and therefore population dynamics) in such long‐lived species because fast‐growing, young individuals increasingly overlap in size with slow‐growing, old individuals (Vogt, 2012).

Here, the use of the rDNA epigenetic clock was tested in known‐age cohorts of European lobsters. Specifically, percentage DNA methylation was quantified at individual rDNA CpGs across rDNA in known‐age, aquaculture‐reared and unknown‐age, wild lobsters using targeted bisulphite sequencing. Elastic Net regression was used to select a subset of loci for predicting chronological age in known‐age lobsters (0–51 months old), and these loci were used to create a penalized regression model for age prediction. The regression model was subsequently used to predict age in wild lobsters estimated to be ≥4 years old. This study is the first to investigate the applicability of site‐specific, DNA methylation‐based markers for age estimation in crustaceans.

## 2 MATERIALS AND METHODS

### 2.1 Study species and sampling

Tissue samples (claw or leg) were obtained from European lobsters of different known ages (0–51 months) reared at the National Lobster Hatchery (NLH) in Cornwall, UK (Table 1). Aquaculture‐reared lobsters ≥7 months old were deployed into sea‐based containers (Daniels et al., 2015) off the coast of Cornwall (UK) ca. 1 month posthatching. The 40‐ and 51‐month‐old lobsters were later recovered from the sea‐based containers (at 36 and 47 months old, respectively) and returned to the hatchery (*n* = 5 and *n* = 17 respectively). Wild lobsters of unknown ages were caught within 12 nautical miles off the coast of Cornwall, were sampled by clipping the terminal end of an antenna (Table 1). All wild‐caught lobsters were above the minimum landing size (MLS) of 88–137 mm carapace length (CL) and therefore estimated to be ≥4 years old. This estimated minimum age is based on size at age data from previous mark–recapture studies (Bannister & Addison, 1998; Schmalenbach et al., 2011; Uglem et al., 2005) in the North‐east Atlantic. The large size range of the wild lobsters suggests they may differ substantially in age (Sheehy et al., 1999).

**TABLE 1:** Sample demography of the 193 European lobsters sequenced in this study

Source	Age at sampling	Age uncertainty	Tissue	Mean CL (mm) (±SD)	*n*
NLH	0.0	0	Claw	NA	27
NLH	1.8	0	Claw	NA	29
NLH	7.3	±0.5 months	Claw	11.2 (2.0)	26
NLH	12.5	±14 days	Claw	16.0 (2.3)	19
NLH	24.8	±14 days	Claw	35.6 (3.1)	32
NLH	40	±14 days	Leg	38.2 (1.83)	5
NLH	51	±14 days	Leg	43.3 (3.70)	17
Wild caught	≥48	Unknown	Antenna	103.9 (15.1)	38

Notes

Ages are time posthatching in months. Error in age estimates arises for individuals that were graded by moult stage rather than hatch date.

Abbreviations: CL, carapace length; SD, standard deviation.

### 2.2 DNA extraction and rDNA reference Sanger sequencing

Genomic DNA was extracted from ca. 2 mm^3^ of tissue excised from within the appendages (claws or antennae) using a salt‐precipitation protocol (modified from Aljanabi & Martinez, 1997) and resuspended in H_2_O. DNA concentration and purity were verified using a NanoDrop 8000 Spectrophotometer (Thermo Scientific).

**FIGURE 1:**
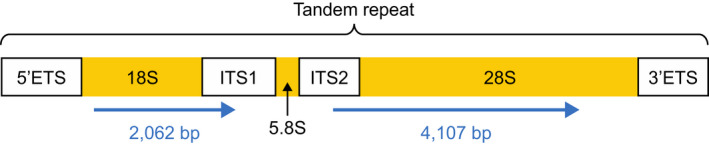
Structure of ribosomal DNA clusters after Dyomin et al. (2016). Blue arrows represent approximate locations of the regions sequenced in this study (total length = 6169 bp) in European lobsters

Ribosomal DNA occurs in tandemly repeated clusters separated by nontranscribed intergenic spacers (Dyomin et al., 2016). Each rDNA cluster comprises three genes essential for ribosome functions (18S, 5.8S and 28S rRNAs), internal transcribed spacers (ITS1 and ITS2) and external transcribed spacers (5′ETS and 3′ETS; Figure 1). Animal rDNA clusters range in length between 8 and 14 kb (Dyomin et al., 2016). Partial sequences for 18S and 28S in *H*. *gammarus* were available in GenBank (Accession numbers: DQ079749 and DQ079789, respectively). To recover additional reference rDNA sequences for *H*. *gammarus*, a combination of published primers and new primers was tested and designed (Table S1). New primers were manually designed using cross‐species alignments of all publicly available rDNA sequences for the European lobster, American lobster (*Homarus americanus*) and Norway lobster (*Nephrops norvegicus*) viewed in AliView (Larsson, 2014). Primer3 software (Rozen & Skaletsky, 2000) was used to ensure compatible annealing temperatures, appropriate GC content (40%–60%), and minimize secondary structures (hairpins) and primer dimer formation.

Polymerase chain reactions (PCRs) were performed in 10 μl, consisting of 5 μl TopTaq Master Mix (Qiagen), 0.2 μl (10 μM) each primer, 4.1 μl ddH_2_O and 0.5 μl DNA. Thermal cycle conditions were initial denaturation at 94℃ for 3 min, followed by 25–30 cycles of denaturation (94℃, 30 s), annealing (30 s) and extension (72℃), with a final extension step at 72℃ for 10 min. Primer‐specific annealing temperatures and extension times are provided in Table S1.

Successful amplification was verified on a 1.5% agarose gel. Amplicons (5 μl) were cleaned with 0.1 μl of Exo1 (Thermo Scientific), 0.2 μl FastAP (Thermo Scientific) and 4.7 μl ddH_2_O at 37℃ for 15 min and 85℃ for 15 min and then sequenced with Sanger sequencing (Eurofins). Sequence chromatograms were viewed in FinchTV (www.digitalworldbiology.com/FinchTV) and poor‐quality regions removed. All available *H*. *gammarus* sequences were subsequently merged in AliView where possible to produce a continuous sequence. The resulting sequences were used as the regions of interest for targeted bisulphite sequencing.

### 2.3 Targeted bisulphite sequencing

Targeted bisulphite sequencing was conducted by Zymo Research including primer design, validation and bioinformatics. Primers were designed to target CpGs across the specified regions of interest with Rosefinch‐Zymo's proprietary primer design tool. Primers were designed so that amplicons were between 100 and 300 bp and primers avoided annealing to CpGs. Where this was not possible, primers were synthesized with a pyrimidine (C or T) at the CpG cytosine in the forward primer, or a purine (A or G) in the reverse primer, to minimize amplification bias to either the methylated or unmethylated allele. Primer sequences are detailed in Table S2. Primers were tested using real‐time PCR (RT‐PCR) with 1 ng of bisulphite‐converted control DNA (from an individual lobster; L312) measured in duplicate. The presence of a single, specific PCR product was confirmed by analysing the RT‐PCR melt curves. RT‐PCRs were deemed successful if the following criteria were met: average crossing point (Cp) values were less than 40, duplicate Cp values did not differ by more than one, the plateau phase was reached before the run ended at cycle 45, melting curves were in the expected range for PCR products, and duplicates had calculated primer melting temperatures within 10% of the coefficient of variation (CV).

Following primer validation, ca. 500ng of lobster DNA from each individual was bisulphite converted using the EZ DNA Methylation‐Lightning™ Kit (Zymo Research). Multiplex amplification of all DNA samples was performed using the Fluidigm Access Array™ System. The resulting amplicons were then pooled and barcoded following the protocol outlined in the Fluidigm Access Array specification sheet (PN 100‐4161 D1). After barcoding, pooled amplicons were purified (ZR‐96 DNA Clean & Concentrator™) and then prepared for sequencing using a MiSeq V2 300 bp Reagent Kit (Illumina) and paired‐end sequencing protocol.

### 2.4 Quantifying percentage CpG methylation

Sequence reads were identified using standard Illumina base‐calling software and then analysed using a Zymo Research proprietary analysis pipeline. Low‐quality reads (Phred score <20) and adapter sequences were removed. Paired‐end sequence reads were aligned back to the reference sequences using Bismark, an aligner designed specifically for bisulphite sequencing data and rDNA methylation calling (Krueger & Andrews, 2011). Primer binding regions were removed from amplicons during rDNA methylation calling. Percentage methylation of each CpG was estimated as the number of reads reporting a C, divided by the total number of reads reporting a C or T, multiplied by 100. Methylated sites with less than ten reads in any individual, or with missing data across individuals, were removed from the data set.

### 2.5 Developing an ageing tool for known‐age lobsters

For lobsters of known age (*n* = 155), the relationship between percentage methylation and age was initially assessed for each quality‐filtered CpG using simple linear regression (‘lm{stats}’: R Core Team, 2017a). A Bonferroni–Holm correction (Holland & Copenhaver, 1987) was applied to control for multiple comparisons using ‘p.adjust{stats}’. Percentage methylation displayed a significant relationship with age at the majority of CpGs (*p* < 0.05; see results Section 3.3). Subsequently, three different multiple regression models were fitted to the data using ‘glmnet{glmnet}’ and the Caret package (Kuhn, 2008) to select the best tuning parameter values, build the final model and evaluate the model performance using cross‐validation (Friedman et al., 2010). A grid of 100 lambda values that ranged between 10^−3^ and 10^3^ was used, and alpha was set at 0 (Ridge), 0.5 (Elastic Net) and 1 (Lasso). Ten cross‐validations were used for the tuning parameters. Models were compared using mean absolute error (MAE), root mean square error (RMSE), *R* ^2^ and the final number of loci in the model.

### 2.6 Assessing the precision of the ageing tool

The ageing model was evaluated in two ways: (i) using age predictions on the known‐age samples that were used in Elastic Net locus selection step. This provides an evaluation of the fit of the model to the data, accepting that overfitting may occur. (ii) To provide an assessment of how the model may work on unknown samples, internal validation using a leave‐one‐out cross‐validation (LOOCV; Picard & Dennis Cook, 1984) was performed. LOOCV involves applying the penalized regression model to all but one individual at a time until all individuals have been left out. Running the Elastic Net model twice—once for evaluation and once for validation—may result in small differences to the chosen loci in each run. Thus, the validation step using LOOCV represents an indication of the precision of the model on unknown data. The validation was run using the same data set as in (i), and using the Caret package to run the cross‐validation using the trainControl(method = ‘LOOCV’) option. Precision was subsequently quantified as the standard deviation (SD) of the mean difference between known and estimated ages. Finally, model precision (based on the LOOCV analysis) was compared among age group cohorts using ‘Anova{car}’ (Fox & Weisberg, 2019).

### 2.7 Effect of sex on age prediction in known‐age lobsters

The sex was known for all 25‐, 40‐ and 51‐month‐old lobsters (*n* = 32, *n* = 5 and *n* = 17, respectively). ‘Anova{car}’ was used to test for differences in predicted age (using the Elastic Net model) between known‐age males and females in each of the 25‐ and 51‐month age cohorts (where sample sizes were sufficient to test for differences in the mean predicted age).

### 2.8 Predicting age in unknown‐age, wild lobsters

Ages for the wild lobsters of unknown age (*n* = 38) were predicted from the Elastic Net model (46 loci) generated in step (i) above and the predict {stats} function.

### 2.9 Relationship between size and (estimated) age

Size (CL) data were available for all aquaculture‐reared lobsters ≥7.3 months old. For these individuals, the relationship between known age and CL was assessed by fitting a von Bertalanffy Growth Model (VBGM) using the FSA package in R (Ogle et al., 2021). The VBGM has been widely applied to decapod crustacean species (Raper & Schneider, 2013). Bootstrapped confidence intervals were estimated using ‘Boot{car}’ (Fox & Weisberg, 2019). It was not possible to fit, the standard VBGM to the wild data as high variability between predicted age and size coupled with poor fit prevented model convergence. Instead, we fitted the Francis parameterization of the VBGM (Francis, 1988), also from the FSA package, which uses fewer parameters in the estimation and converged.

### 2.10 Statistical analysis

All statistical analyses were performed in R version 3.6.3 (R Core Team, 2020) using RStudio version 1.2.5003 (RStudio Team, 2016). Plots were produced using ‘ggplot{ggplot2}’ (Wickham, 2016) or ‘Plot{graphics}’ (R Core Team, 2017b). For independent samples *t* tests, normality and variance were assessed using ‘shapiro.test {stats}’ and ‘leveneTest{car}’, respectively. ‘shapiro.test {stats}’ was also used to check normality for Pearson's correlations.

## 3 RESULTS

### 3.1 rDNA Sanger sequencing

Two continuous reference sequences spanning partial 18S through to the start of ITS1 (2062 bp) and the end of ITS2 through to partial 28S (4107 bp) were generated (Figure 1). These two sequences were used as the regions of interest for bisulphite sequencing. Sequences for the gap between the two regions (end of ITS1 to the beginning of ITS2) were not possible to obtain despite testing published ITS1 (Chu et al., 2001) and ITS2 primers (Harris & Crandall, 2000), and primers designed in this study (Gam_ITS1_F: 5′‐AGTCGTAACAAGGTTTCCGT‐3′ and Gam_ITS2_R: 5′‐TCTTCACCACCGACATTACCA‐3′), possibly because of intra‐individual variation in ITS sequences (Bower et al., 2009; Harris & Crandall, 2000).

### 3.2 Bisulphite sequencing quality control

Bisulphite conversion rates were greater than 99% for each DNA sample. Amplicons were successfully generated from bisulphite‐converted DNA for 5154 bp across the two regions (84% of combined length; Figure S1; Table S1). A total of 436 CpGs were sequenced, and 355 were retained for downstream analyses following removal of sites that were not successfully sequenced across all individuals or had fewer than ten reads in any individual. Average read coverage per individual across the 355 loci was 7107 for the 0‐ to 25‐month cohorts and the wild and 25,253 for the 40‐ and 51‐month cohorts (which were sequenced in a second run).

### 3.3 Age prediction using CpG methylation in known‐age lobsters

Simple linear regressions showed that percentage methylation had a significant relationship with age at 60% of filtered CpGs (*n* = 214/355; Bonferroni–Holm corrected *p* < 0.05). Comparing the model performance of the three models investigated (Ridge, Lasso and Elastic Net), using MAE, RMSE and *R* ^2^, both the Elastic Net and Lasso models had lower error (MAE and RMSE) and a higher *R* ^2^ than the Ridge model (Figure S2a). However, the Lasso and Elastic Net models were very similar in all estimates and we selected Elastic Net for the final model as it used fewer loci overall and had the lowest median MAE with an almost identical *R* ^2^.

**FIGURE 2:**
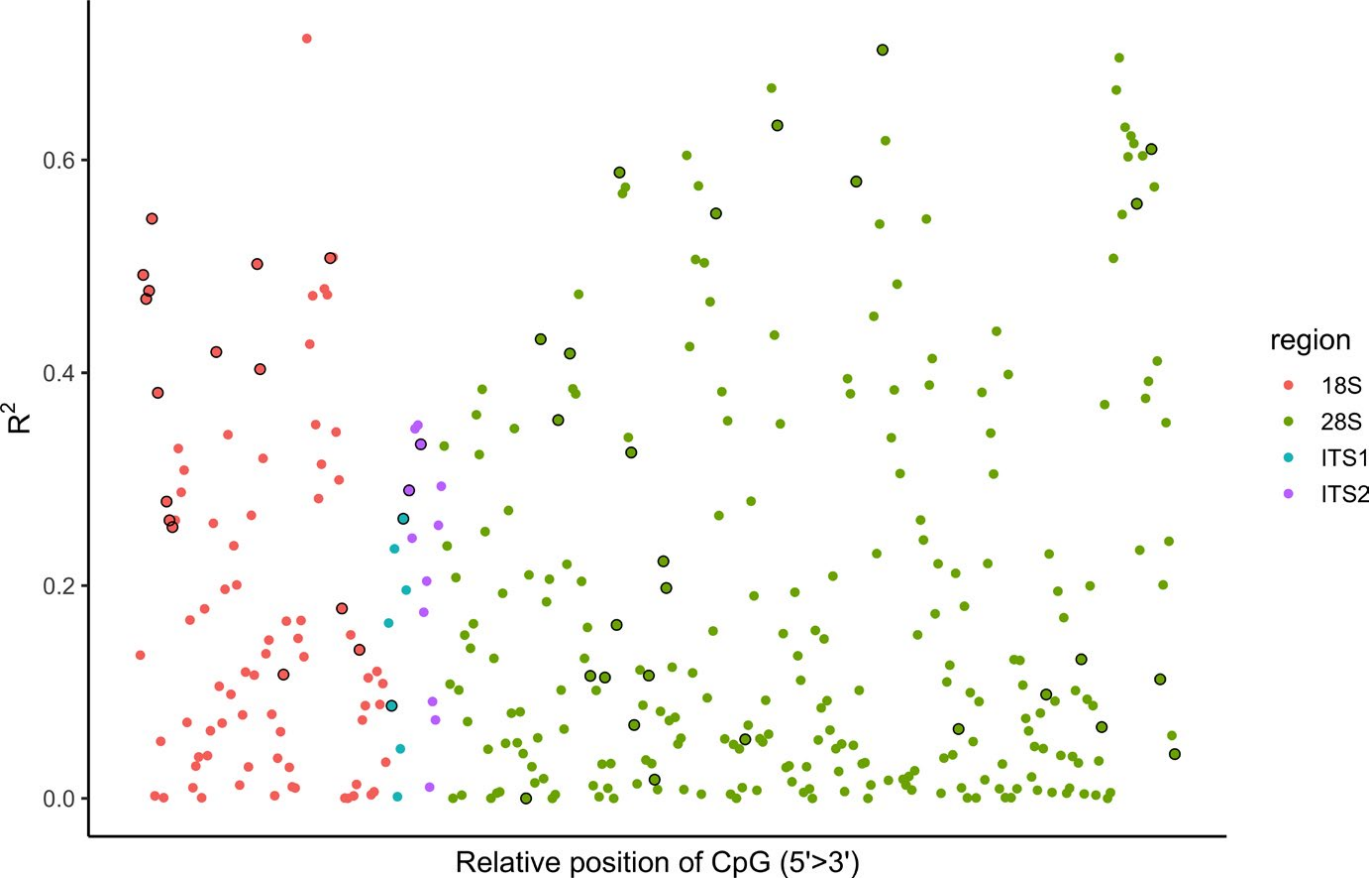
Regression coefficients from linear regressions of percentage methylation and age for 355 rDNA CpGs in 155 known‐age European lobsters (0–51 months old). Sites ordered according to their relative position along the rDNA region of interest (ROI) and coloured by gene region. Black outlined circles show the 46 loci selected by the Elastic Net regression for the ageing model

**FIGURE 3:**
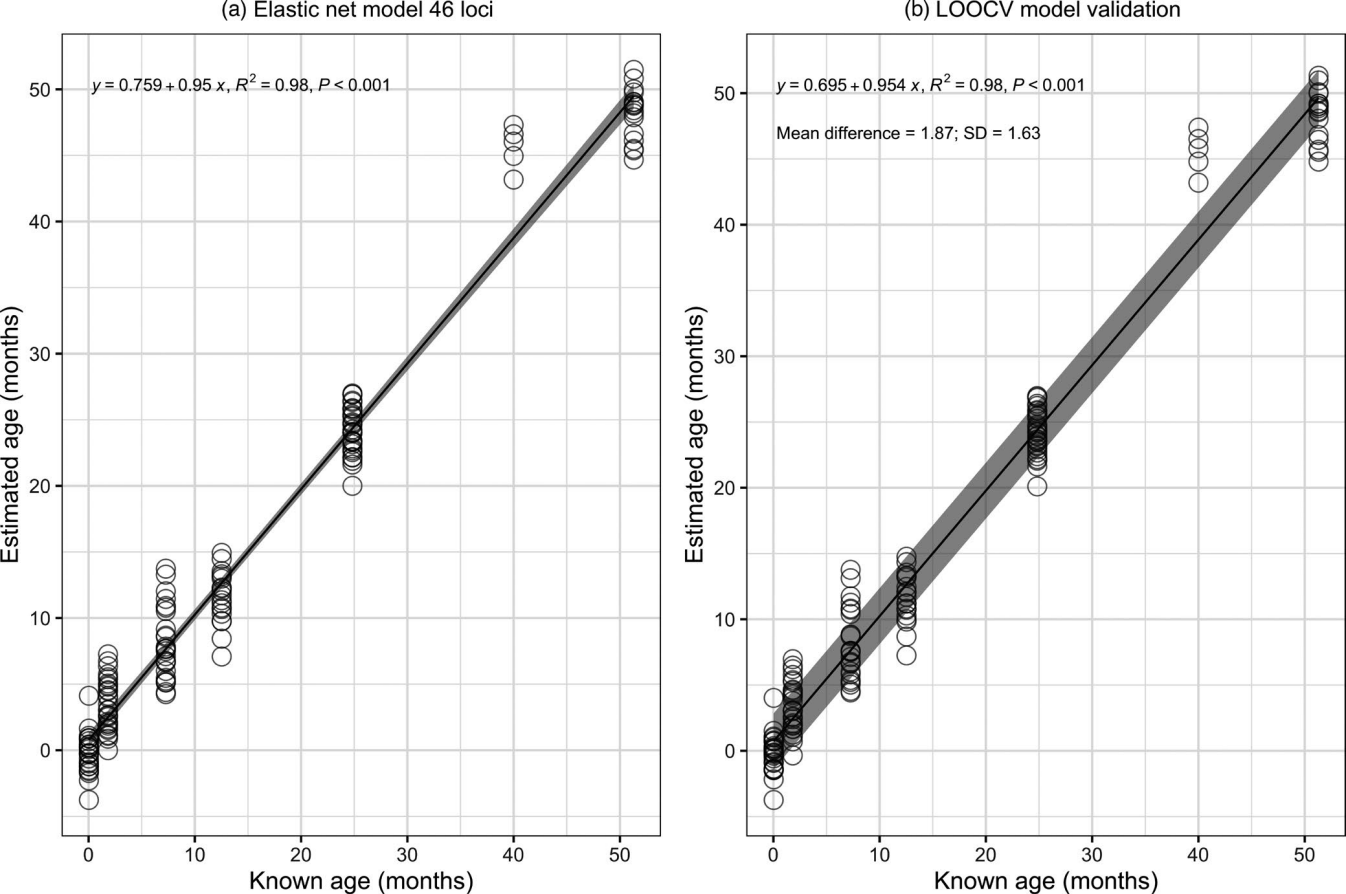
(a) Elastic Net regression for estimated age based on percentage methylation at 46 CpG loci in 155 known‐age European lobsters. (b) Precision of the model as determined using a leave‐one‐out cross‐validation analysis (LOOCV). Grey regions represent the 95% confidence intervals for the regression line in plot a and represent the mean ± qnorm (0.975) × SD/sqrt(*n*) of the difference between known and predicted age in plot b

Forty‐six of the 355 CpGs were included in the Elastic Net model (Table 2; Figure S2b). The age‐related CpGs were relatively evenly distributed along the rDNA (Figure 2). Estimated age according to percentage methylation across the 46 loci had a highly significant relationship with actual age (*p* < 0.001), explaining 98% of the variation in chronological age using the test data set and predictions from the Elastic Net model (*R* ^2^ = 0.98; Figure 3a).

**TABLE 2:** The 46 CpGs with nonzero coefficients in the Elastic Net regression model, which assessed the relationship between percentage methylation and lobster age in 155 European lobsters (0–51 months old) at 355 rDNA CpGs

Gene	Position	Elastic Net coefficient	*R* ^2^	Adjusted *p*
18S	231	11.342	0.492	<0.001
18S	235	5.044	0.469	<0.001
18S	242	23.531	0.477	<0.001
18S	247	9.118	0.545	<0.001
18S	253	4.319	0.381	<0.001
18S	318	−3.579	0.279	<0.001
18S	325	−18.713	0.261	<0.001
18S	340	−60.333	0.255	<0.001
18S	631	25.945	0.420	<0.001
18S	904	−5.237	0.502	<0.001
18S	914	−1.576	0.403	<0.001
18S	1026	21.638	0.116	0.003
18S	1304	−17.085	0.508	<0.001
18S	1595	6.893	0.179	<0.001
18S	1667	−33.717	0.140	<0.001
ITS1	1793	42.097	0.087	0.028
ITS1	1874	−13.192	0.263	<0.001
ITS2	249	−41.176	0.290	<0.001
ITS2	275	−14.155	0.333	<0.001
28S	969	6.140	0.000	ns
28S	992	60.321	0.432	<0.001
28S	1029	−10.776	0.356	<0.001
28S	1057	−33.947	0.418	<0.001
28S	1116	19.951	0.115	0.003
28S	1167	−6.639	0.114	0.003
28S	1202	−14.738	0.163	<0.001
28S	1214	−1.657	0.588	<0.001
28S	1303	−15.052	0.325	<0.001
28S	1307	−4.046	0.069	ns
28S	1358	2.400	0.115	0.003
28S	1384	0.461	0.018	ns
28S	1413	−1.400	0.223	<0.001
28S	1423	−0.116	0.198	<0.001
28S	1568	0.890	0.550	<0.001
28S	1710	−1.082	0.056	ns
28S	2154	−20.514	0.633	<0.001
28S	2656	107.191	0.580	<0.001
28S	2761	27.169	0.704	<0.001
28S	3048	30.569	0.065	ns
28S	3538	1.622	0.098	0.012

Notes

*R*^2^ and *p*‐values from simple linear regression of percentage methylation with lobster age for each CpG. A Bonferroni–Holm correction was applied to all *p*‐values. Positions according to the reference sequences compiled in this study.

### 3.4 Ageing model precision

The LOOCV analysis was run using the same parameters as the Elastic Net model and selected 48 loci in comparison with the 46 selected by Elastic Net (with 45 loci in common). The estimated precision of the ageing model was 1.63 months—the SD of the mean difference between known and estimated ages (Figure 3b). The mean of the age estimates was significantly different among age groups (ANOVA: *F*_(6,153)_ = 9.525; *p* < 0.0001; Figure S3), and this was driven almost entirely by the 40‐month cohort (*n* = 5) in which predicted ages were significantly higher than actual ages for all pairwise comparisons (Tukey's post hoc test *p* < 0.001; Table S3).

### 3.5 Effect of sex on age prediction in known‐age lobsters

**FIGURE 4:**
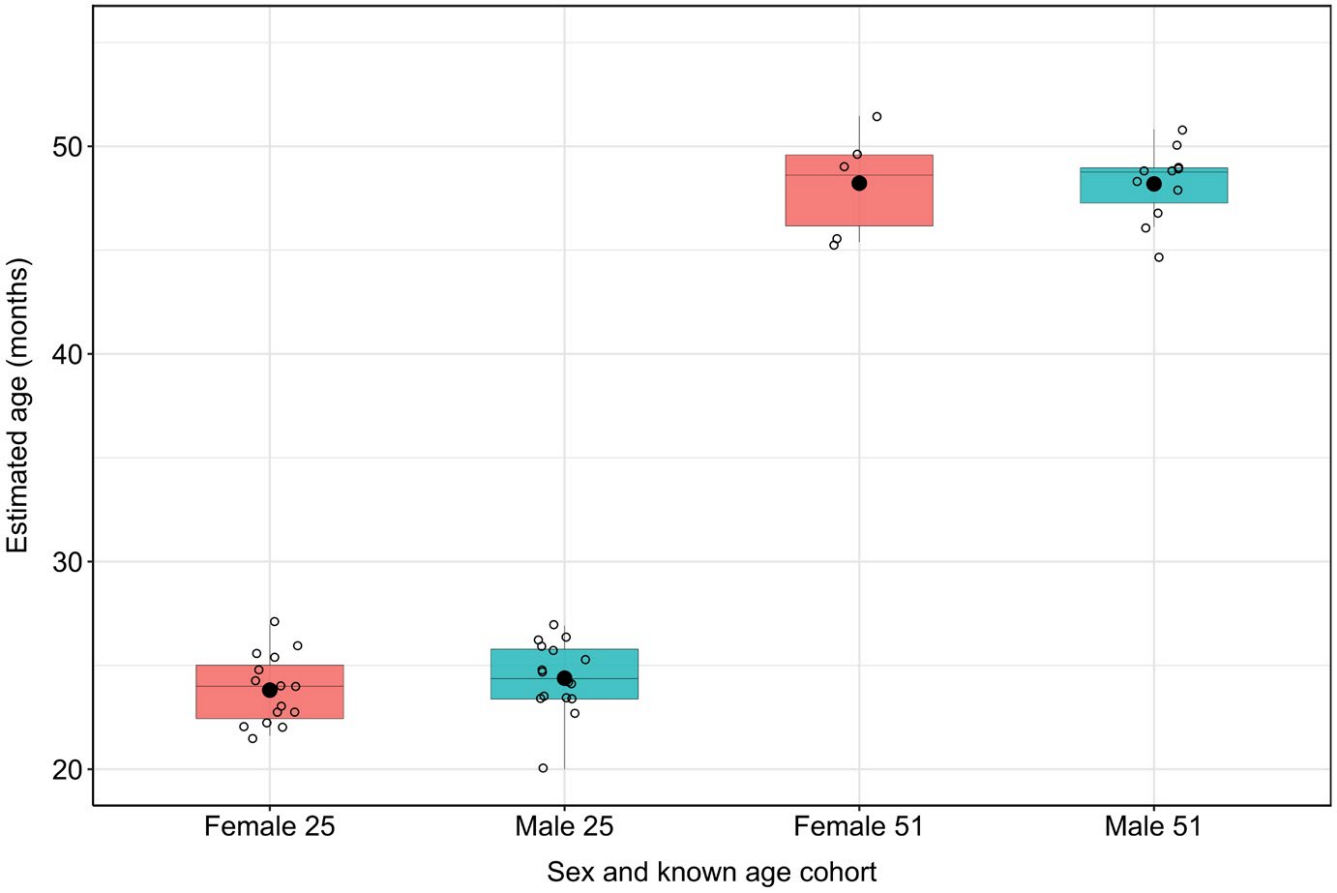
Predicted European lobster age using the 46 locus Elastic Net model in males and females of the 25‐ and 51‐month cohorts

Methylation levels were compared between the sexes in lobster cohorts that were 25 and 51 months old (the only cohorts in which sufficient numbers of individuals were sexed for comparison). In the 25‐month cohort, females had higher methylation than males at 44 out of the 46 loci used (ten tests were significant at an uncorrected *p* < 0.05; none of the pairwise tests were significantly different from zero after Bonferroni–Holm correction; Table S4). In the 51‐month cohort, males had higher methylation that females at 45 out of the 46 loci used (two tests were significant at an uncorrected *p* < 0.05, none of the pairwise tests was significantly different from zero after Bonferroni–Holm correction; Table S5). Males and females did not differ in estimated age while controlling for cohort age using the 46 locus Elastic Net model (ANOVA: *F*_(1,49)_ = 0.414; *p* = 0.523; Figure 4).

### 3.6 Predicting age in unknown‐age wild lobsters

**FIGURE 5:**
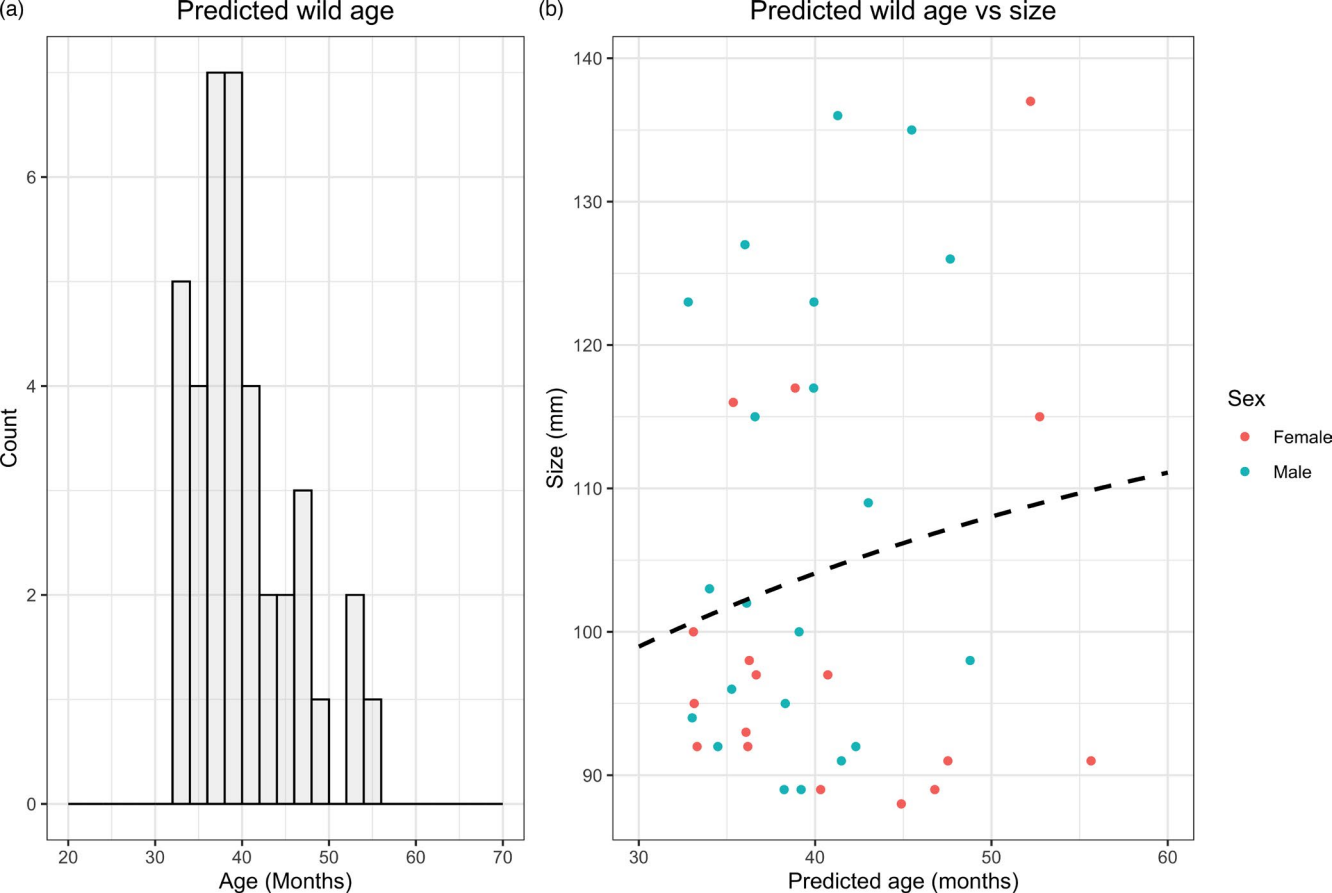
(a) Histogram of predicted age in wild European lobsters using the Elastic Net 46 locus model. (b) Predicted age vs carapace size (mm) of wild caught lobsters. The dotted line shows the Francis implementation of the von Bertalanffy Growth Model

The ages of unknown‐age, wild lobsters were predicted using the 46 locus Elastic Net model. Wild lobsters were estimated to have a mean age of 40.1 months (Range = 32.8–55.7 months; Figure 5a).

### 3.7 Relationship between carapace size (SL) and known and estimated age

The best fitting von Bertalanffy Growth Model was CL = 46.2(1‐e^−0.06(age − 3.288)^ for the known‐age data (Figure S6). There was a poor model fit of the Francis parameterization of the VBGM using wild size and predicted age (Figure 5b).

### 3.8 Investigating a reduced locus model

To investigate the utility of an ageing model with fewer loci, we selected the top fifteen loci based on *R^2^* from the Elastic Net model and built a reduced locus ageing model using ‘lm{stats}’. This reduced model had a highly significant *R* ^2^ of 0.95 (*p* < 0.001; Figure S5a). A LOOCV validation demonstrated that the 15 loci model is slightly less accurate than the 46 locus model (SD = 1.6 and 2.13 months, respectively; Figure S5b). Predicted ages for the wild lobsters based on the full 46 locus model and the reduced 15 locus model were highly correlated (*R* ^2^ = 0.89; *p* < 0.001; Figure S7).

## 4 DISCUSSION

This study investigated whether patterns of CpG methylation in rDNA could be used to estimate age in European lobsters. In European lobsters ranging in known age from 0 to 51 months, percentage methylation had a significant relationship with chronological age at a large number of the sequenced loci (*n* = 214, 60% of CpGs that passed quality control). Forty‐six loci were selected by Elastic Net regression on known‐age lobsters and validated using LOOCV. The ageing model predicted lobster age with high accuracy (*R* ^2^ = 0.98; *p* < 0.001) and precision (SD = 1.6 months). The ageing model was then used to predict age in wild lobsters of unknown age which resulted in an estimated mean age of 40.1 months old (range = 32.8–55.7 months). The accuracy of the model for known‐age individuals is among the highest reported in any animal, with the average *r*/*R* ^2^ for epigenetic clocks developed in other animals, based on alternative regions of the genome, being 0.74 (Range = 0.58–0.95; Beal et al., 2019; Bocklandt et al., 2011; De Paoli‐Iseppi et al., 2019; Han et al., 2018; Ito et al., 2018; Polanowski et al., 2014; Wright et al., 2018). These results suggest that the measurement of rDNA methylation changes holds considerable promise as a cost‐effective marker of age in European lobsters and supports the hypothesis that rDNA may harbour an evolutionarily conserved clock of animal age (Wang & Lemos, 2019). Further work is required to test the assay on older, known‐age European lobsters and across different tissue types.

Patterns of DNA methylation have been well characterized in various vertebrates, and locus‐specific methylation patterns have been used to determine chronological and biological age in a growing number of vertebrate species (De Paoli‐Iseppi et al., 2019; Horvath, 2013; Robeck et al., 2021; Wilkinson et al., 2021). In comparison, relatively limited research has been conducted on methylation in invertebrates (Roberts & Gavery, 2012). Vertebrate genomes are almost always highly methylated, whereas invertebrate genomes appear to be far more variable. Some invertebrate species have no detectable cytosine methylation, although they do have adenine methylation (e.g. *Caenorhabditis elegans*: Greer et al., 2015), or only negligible amounts during specific developmental stages (e.g. *Drosophila melanogaster*: Lyko et al., 2000), whereas other invertebrates have intermediate levels of DNA methylation (e.g. *Ciona intestinalis*: Suzuki et al., 2007). As of yet, DNA methylation has been studied in just a handful of crustacea, with global levels ranging from no detectable methylation in Artemia (Warner & Bagshaw, 1984), 0.05% in prawns (*Macrobrachium rosenbergii*: Feng et al., 2007) to 3% in crayfish (*Procambarus fallax*: Vogt et al., 2015). This limited research suggests that methylation is generally low in crustaceans compared with other animals but highly variable among species (Vandegehuchte et al., 2009). Less still is known about age‐related patterns of methylation in crustaceans; a recent study found no evidence for age‐dependent global methylation changes in two crayfish species (*P*. *fallax* and marbled crayfish), but this was based on two age groups (juveniles vs. adults) with very small sample sizes per group (Vogt et al., 2015). Thus, this study represents one of the first studies to investigate the relationship between methylation levels and age in an invertebrate system.

The age range of individuals used to calibrate the ageing assay represents a small proportion of the maximum estimated lifespan for European lobsters (42–72 years; Sheehy et al., 1999). Here, the oldest, known‐age lobsters were 51 months old. Obtaining tissue samples across a broad range of known ages is extremely difficult for this long‐lived species, largely because they are harvested from the wild (not farmed) and cannot easily be individually tagged for recapture studies (due to moulting). This limitation (the lack of known‐age individuals) will apply to most studies involving economically important crustaceans, which often have long lifespans (Vogt, 2019). However, obtaining older, known‐age European lobster DNA should be possible in the future with further repeated sampling of lobsters. Extending the ageing assay to cover a larger range of known lobster ages would be of particular interest to fisheries management, as the maximum known‐age samples used in the current study are close to the minimum estimated age for lobsters exploited by fisheries.

To investigate the ageing model's ability to age older lobsters, particularly without having exact, known‐age individuals, the model was used to predict the ages of wild lobsters of unknown age. This resulted in an average estimated age of 40.1 months (range = 32.8–55.7 months). Based on the size of the wild lobsters, we expect minimum ages to be between 4 and 9 years old. Therefore, the predicted ages from the methylation assay are not unrealistic, although at the lower end of the expected range for the larger individuals. The most reliable information on size at age for European lobsters in the UK comes from a recapture study in which thousands of hatchery‐reared, juvenile (stage VII) lobsters were microwire tagged, released from Bridlington on the East coast of the UK, and subsequently recaptured (Bannister & Addison, 1998). Unfortunately, tissue samples were not taken as part of this study. However, tagged lobsters reached MLS (88 mm) 4–9 years after release, suggesting that the wild lobsters in our study are at least this old based on their size (88–137 mm CL). Similar patterns of growth were observed in recapture studies in other sites around the UK (numbers unreported: Bannister & Addison, 1998), Heligoland Archipelago off the north‐west coast of Germany (MLS = 85 mm CL at 4–7 years: Schmalenbach et al., 2011) and Norway (MLS = 88 mm CL at 4–8 years: Uglem et al., 2005). These studies suggest that the minimum age at which lobsters reach legal size shows little population dependency, although none of the studies were based in Cornwall where lobster growth may be faster as a result of environmental differences (e.g. higher water temperature; Ellis et al., 2015). Here, we are also extrapolating beyond the range of the regression models (calibrated using younger, known‐age lobsters) which is potentially risky. However, without known‐age lobsters spanning the entire lifespan of up to 70 years, this is almost impossible to avoid. It is worth noting that the sizes of the known‐age lobsters that were raised in sea cages and then subsequently returned to the hatchery have smaller size at ages than has been observed in previous mark–recapture studies (Figure S6). This may be due to several reasons, but perhaps most importantly, food availability and variety are likely to be lower in semi‐confined systems compared with a truly wild environment.

There are a number of factors that should be considered when attempting to age wild animals using epigenetic clocks—especially when estimating the age of individuals that may be older than those used to calibrate the model. Firstly, epigenetic clocks may fail to provide accurate estimates of age in older individuals if the CpGs used reach saturation before old age. In other words, if loci become fully methylated or completely unmethylated early in life, based on single locus linear regressions of percentage methylation against known age, the average time to saturation of the 46 loci used in the ageing model is 40.1 years with a minimum of 84.4 months (9.7 years) and a maximum of 1782.9 months (148 years; excluding one extreme outlier, locus 28S_969; Table S6). The average value is lower than the maximum estimated known age of European lobsters (70 years), but the range of saturation ages appears appropriate for the lifespan of the study organism.

Another potential cause of error in wild lobster age estimation is that rDNA methylation changes may be nonlinear with age and reach a plateau phase (irrespective of saturation). Previous studies on epigenetic changes have primarily shown linear trends across wide age ranges in a number of different species, tissues and genomic regions (Anastasiadi & Piferrer, 2020; Bocklandt et al., 2011; Ito et al., 2018; Polanowski et al., 2014). Data from human studies suggest nonlinear epigenetic changes do exist, although such trends are restricted to early life (Horvath, 2013; Snir et al., 2019). Specifically, human DNA methylation has been shown to change at an accelerated rate in young individuals (birth to adolescents) and thereafter varies in a decelerated, linear fashion from early adulthood through to old age (Horvath, 2013; Snir et al., 2019). During the adulthood phase of the Horvath epigenetic clock, predicted ages increased at the same rate as chronological age on average (Horvath, 2013). Such trends of early acceleration followed by deceleration are best described by a logarithmic function with age (Snir et al., 2019). If a similar pattern occurs in European lobsters, a linear regression equation would result in an overestimation of age in older individuals, which does not appear to be the case in this study. It is more likely that the predicted ages of wild lobsters are being underestimated, based on previous tagging studies.

An additional cause of model inaccuracy may occur because of tissue‐dependent differences in epigenetic change. Claws and antennae were sampled from known‐age and wild lobsters, respectively. If rDNA methylation levels change at different rates across tissues, ageing models should be trained for different tissue types. Tissue‐dependent patterns of age‐related CpG methylation have been observed in humans (Christensen et al., 2009; Horvath, 2013), mice (Maegawa et al., 2010; Spiers et al., 2016) and fish (Anastasiadi & Piferrer, 2020). For example, in European sea bass (*Dicentrarchus labrax*) an epigenetic predictor of age created using muscle DNA was found to perform well in the testis but failed to accurately predict age from ovary tissue (Anastasiadi & Piferrer, 2020). On the other hand, methylation status at some loci allows for multitissue predictors of age. ‘Horvath's clock’, for example, can accurately estimate human age from any of 51 different tissue and cell types based on the weighted average of 353 CpGs (Horvath, 2013). Multitissue predictors tend to come at the cost of requiring more CpGs to capture the variation across tissues (Horvath, 2013; Stubbs et al., 2017). Antennae were collected from wild lobsters as these can be sampled nondestructively, and without compromising the commercial value of the catch by removing a claw. Other nondestructive tissue samples include legs and pleopods, which lobsters can autotomize and regrow (Butler, 2017). Thus, further work is required to characterize variation in methylation signal in different lobster tissues.

Male and female lobsters did not differ significantly in percentage methylation at the loci included in the ageing model or in estimated age overall across the 25‐ and 51‐month‐old cohorts. Individual loci were also investigated for sex‐related differences. In the 25‐month cohort, females had higher methylation than males at 44 out of the 46 loci used (ten tests were significant at an uncorrected *p *< 0.05; none of the pairwise tests were significantly different from zero after Bonferroni–Holm correction). In the 51‐month cohort, males had higher methylation that females at 45 out of the 46 loci used (two tests were significant at an uncorrected *p *< 0.05, none of the pairwise tests was significantly different from zero after Bonferroni–Holm correction). Sex‐related differences in methylation at age‐related CpGs have been reported from bottlenose dolphins (Beal et al., 2019) and short‐tailed shearwaters (De Paoli‐Iseppi et al., 2019) but only for a portion of the CpGs investigated and these differences did not affect the multiple regression ageing models. None of the age‐related CpGs investigated in humpback whales displayed sex‐specific regressions (Polanowski et al., 2014). These results suggest that sex‐dependent DNA methylation is context specific. Future work should investigate whether differences in rDNA methylation occur across European lobsters of different known ages. If differences exist, and sex accounts for unexplained variation in the ageing model, sex‐specific regressions could improve the accuracy of epigenetic age estimation in lobsters.

Ultimately, any ageing model developed for commercially exploited crustaceans should be quick and easy to use, and relatively cheap if it is to be applied to fisheries management. This aim will be facilitated by developing an assay that has as few loci as possible, but with maximum power. We tested a model which used fifteen of the best performing loci (based on *R* ^2^) and this resulted in a model with very high predictive ability (*R* ^2^ = 0.95; SD = 2.13 months; *p* < 0.001), but not quite as high as the 46 locus model (*R* ^2^ = 0.98; SD = 1.6 months; *p* < 0.001). This demonstrates the potential of small panels of methylation‐based markers for assisting in age‐class assignment in fisheries management.

Finally, some variation was found among known‐age cohorts in terms of their fit to the ageing model. The 40‐month cohort appeared to be ‘overaged’ in comparison with the ageing model (Figure S3). This could be an artefact of the small sample size (*n* = 5) for this cohort, or potentially a technical artefact associated with a second run of the laboratory analysis. We would recommend running a range of known‐age internal standards on each repeated batch to minimize technical bias among different batches of individuals.

In conclusion, this study is the first to investigate the rDNA epigenetic clock of ageing in wild animal and suggests that this method holds considerable promise as an ageing tool for European lobsters. Further development and validation of this work is needed before the method can be applied for use in fisheries science. As an area of priority, the rDNA ageing assay requires testing across a wider age range of European lobsters, and with a comparison of the effect of tissue types and environment. Such information will help to shed light on the main unanswered questions presented here: are age‐related patterns of rDNA methylation (a) linear into adulthood and/or (b) tissue‐specific? Information on whether the rDNA epigenetic clock is sex or population dependent will also be of value before such a tool can be widely adopted. Finally, because of the highly conserved nature of the ribosomal DNA, there is the theoretical possibility of applying the loci developed within this study to other crustacean species where establishing chronological age is an issue (although this has yet to be tested).

## CONFLICT OF INTEREST

The authors have declared no conflict of interest.

## Supporting information

Table S2

Supplementary Material

## Data Availability

The data that support the findings of this study are openly available in Dryad Digital Repository at https://doi.org/10.5061/dryad.8kprr4xp5.
