# Supplementary material for: Ageing European lobsters (Homarus gammarus) using DNA methylation of evolutionarily conserved ribosomal DNA
Source: Evol Appl. 2021 Sep 23;14(9):2305–18. doi: 10.1111/eva.13296 (PMC8477595; doi:10.1111/eva.13296)
Supplement: Supplementary file 2 — Supplementary Material [file EVA-14-2305-s002.docx]

**Supporting information**

**Title:** Ageing European lobsters (*Homarus gammarus*) using DNA methylation of evolutionarily-conserved ribosomal DNA

**Table S1** Primers used to PCR amplify ribosomal DNA in European lobsters.

| Primer name | Primer sequence (5’ → 3’) | Target | Annealing (^o^C) | Extension (min) | | *n* cycles | Reference |
| --- | --- | --- | --- | --- | --- | --- | --- |
| ITS1_fwd_SP-1-5 | CACACCGCCCGTCGCTACTA | 18S:ITS1 | 60 | | 1 | 30 | Chu et al. 2001 |
| ITS2_rev_SP-1-3 | ATTTAGCTGCGGTCTTCATC |  |  |  |  |  | Chu et al. 2001 |
| ITS1_fwd_SP-1-5 | CACACCGCCCGTCGCTACTA | ITS2:28S | 60 | | 2 | 25 | Chu et al. 2001 |
| 28S_rev_PT3 | TTCAGTCGCCCTTACTAAGGGAATCC |  |  |  |  |  | Tang et al. 2003 |
| 28S_gam_1F | GGTTATCCCAGGCAGCATTG | 28S | 55 | | 1 | 25 | This study |
| 28S_gam_2R | AGTCATAGTTACTCCCGCCG |  |  |  |  |  | This study |
| 28S_gam_3F | CGGCGGGAGTAACTATGACT | 28S | 60 | | 1 | 25 | This study |
| 28S_gam_4R | ATCGATAGGCCTTGCTTTCG |  |  |  |  |  | This study |

*Note:* All primers were successfully used for subsequent Sanger sequencing except ITS2_rev_SP-1-3, which failed to produce clean sequence despite several attempts at optimisation.

a) 18S–ITS1

**
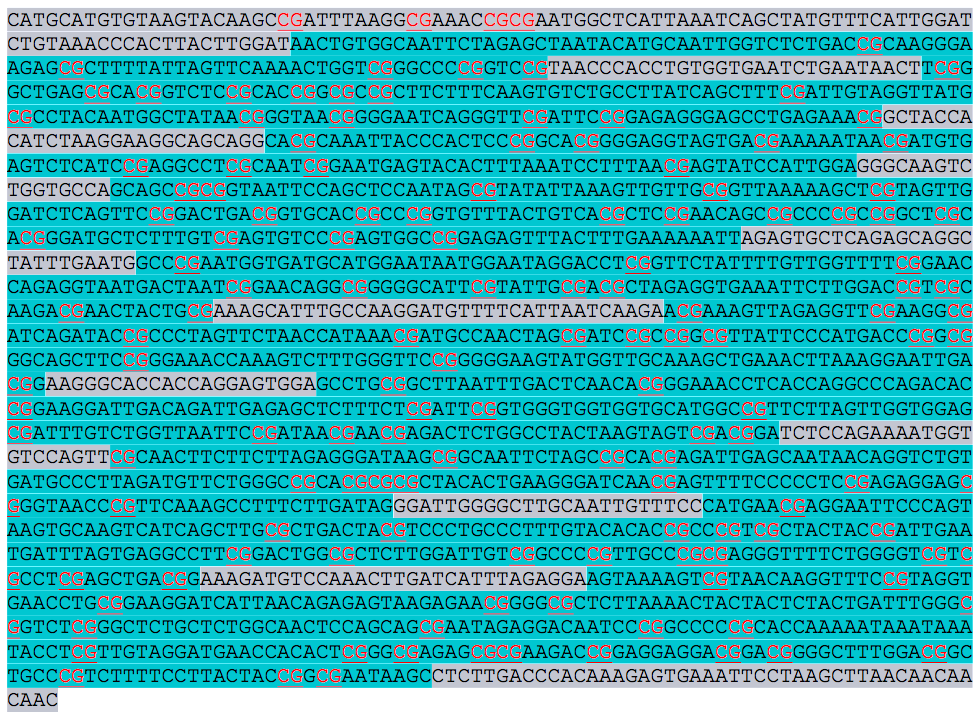
**

b) ITS2–28S

**
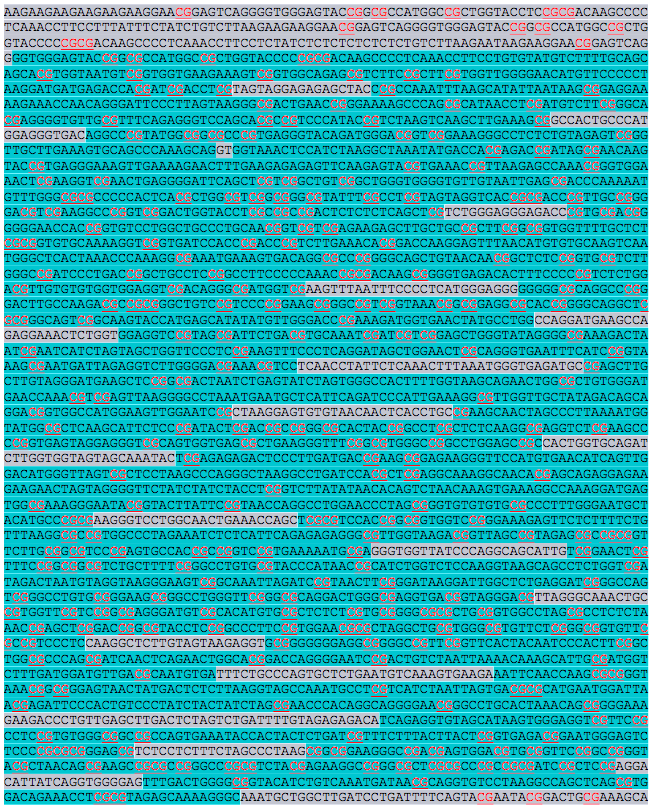
**

**Figure S1** Sequences of the two ribosomal DNA regions of interest (a and b). Regions successfully covered by amplicons for bisulphite sequencing in blue and regions not tested in grey. CpG dinucleotides in red.


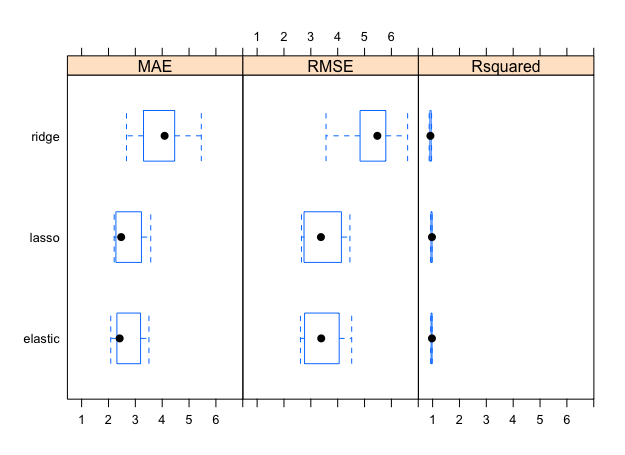


Figure S2a. Boxplot of the error and fit of the three ageing models investigated (Ridge, Elastic Net and Lasso). MAE=Mean Absolute Error; RMSE=Root Mean Squared Error, R squared = R squared.

**
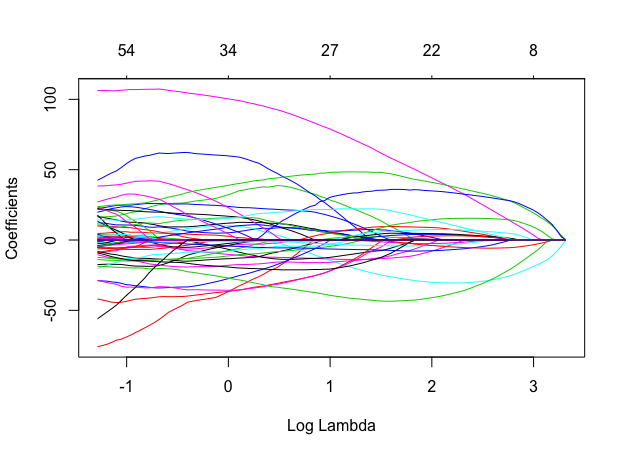
**

**Figure S2b** Elastic Net regression coefficient pathways for the relationship between percentage methylation at individual CpGs (*n* = 355) and lobster age at different levels of log lambda. The top axis represents the number of predictor variables with non-zero coefficients.


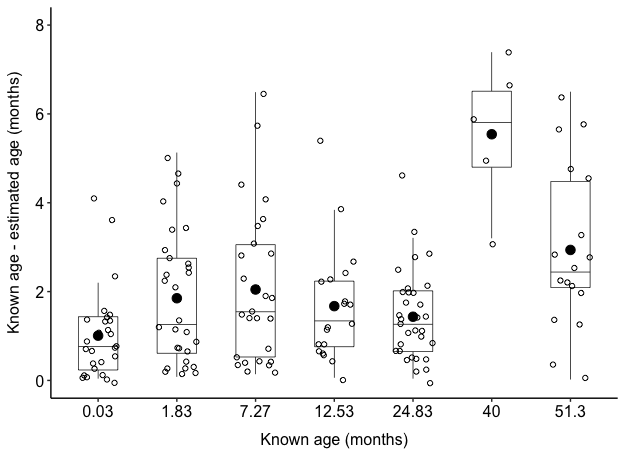


**Figure S3** Differences between known and estimated ages across seven age groups of European lobster. Boxplots display a mean dot (solid black point), median line, inter-quartile range (IQR) boxes, 1.5*IQR whiskers, and data points.

**Table S3** Tukey’s post-hoc tests of difference in mean predicted error between known age and predicted age in the different age (in months) compared. Significant contrasts (*p* < 0.05) in bold and underlined.

| Contrast | Estimate | SE | df | t.ratio | p.value |
| --- | --- | --- | --- | --- | --- |
| 0-2 | -0.843 | 0.379 | 148 | -2.226 | 0.2883 |
| 0-7 | -1.039 | 0.389 | 148 | -2.672 | 0.1129 |
| 0-13 | -0.665 | 0.424 | 148 | -1.568 | 0.7025 |
| 0-25 | -0.426 | 0.37 | 148 | -1.152 | 0.9104 |
| **0-40** | **-4.534** | **0.689** | **148** | **-6.579** | **<0.001** |
| **0-51** | **-1.93** | **0.438** | **148** | **-4.403** | **<0.001** |
| 2-7 | -0.197 | 0.382 | 148 | -0.514 | 0.9986 |
| 2-13 | 0.178 | 0.418 | 148 | 0.425 | 0.9995 |
| 2-25 | 0.416 | 0.363 | 148 | 1.147 | 0.9123 |
| **2-40** | **-3.692** | **0.685** | **148** | **-5.386** | **<0.001** |
| 2-51 | -1.087 | 0.432 | 148 | -2.515 | 0.1616 |
| 7-13 | 0.374 | 0.427 | 148 | 0.876 | 0.9756 |
| 7-25 | 0.613 | 0.374 | 148 | 1.64 | 0.6569 |
| **7-40** | **-3.495** | **0.691** | **148** | **-5.056** | **<0.001** |
| 7-51 | -0.891 | 0.442 | 148 | -2.017 | 0.4082 |
| 13-25 | 0.239 | 0.41 | 148 | 0.582 | 0.9972 |
| **13-40** | **-3.869** | **0.711** | **148** | **-5.438** | **<0.001** |
| 13-51 | -1.265 | 0.473 | 148 | -2.677 | 0.1115 |
| **25-40** | **-4.108** | **0.681** | **148** | **-6.035** | **<0.001** |
| **25-51** | **-1.504** | **0.425** | **148** | **-3.539** | **0.001** |
| **40-51** | **2.604** | **0.72** | **148** | **3.616** | **0.007** |

**Table S4.** Mean methylation percentage at each locus, raw and Bonferroni–Holm corrected *p*-values from independent samples *t*-test between sexes in the 25 month age cohort. Significant *p*-values (< 0.05) in bold and underlined.

| Locus | Mean female methylation | Mean male methylation | raw p-value | | BH corrected p-value | |
| --- | --- | --- | --- | --- | --- | --- |
| 18S_231 | 0.837 | 0.828 | 0.120 | | 0.263 | |
| 18S_235 | 0.724 | 0.709 | 0.143 | | 0.272 | |
| 18S_242 | 0.865 | 0.859 | 0.185 | | 0.304 | |
| 18S_247 | 0.629 | 0.605 | **0.043** | | 0.199 | |
| 18S_253 | 0.741 | 0.729 | 0.134 | | 0.272 | |
| 18S_318 | 0.298 | 0.278 | 0.087 | | 0.263 | |
| 18S_325 | 0.331 | 0.313 | 0.095 | | 0.263 | |
| 18S_340 | 0.051 | 0.048 | 0.396 | | 0.520 | |
| 18S_631 | 0.607 | 0.591 | 0.206 | | 0.312 | |
| 18S_904 | 0.352 | 0.326 | **0.036** | | 0.199 | |
| 18S_914 | 0.437 | 0.410 | **0.033** | | 0.199 | |
| 18S_1026 | 0.422 | 0.401 | 0.052 | | 0.199 | |
| 18S_1304 | 0.214 | 0.198 | **0.046** | | 0.199 | |
| 18S_1595 | 0.739 | 0.730 | 0.205 | | 0.312 | |
| 18S_1667 | 0.579 | 0.549 | **0.014** | | 0.199 | |
| ITS1_1793 | 0.821 | 0.819 | 0.843 | | 0.862 | |
| ITS1_1874 | 0.225 | 0.204 | **0.026** | | 0.199 | |
| ITS2_249 | 0.190 | 0.176 | **0.047** | | 0.199 | |
| ITS2_275 | 0.209 | 0.197 | 0.153 | | 0.272 | |
| 28S_969 | 0.637 | 0.624 | 0.183 | | 0.304 | |
| 28S_992 | 0.151 | 0.144 | 0.143 | | 0.272 | |
| 28S_1029 | 0.296 | 0.278 | 0.051 | | 0.199 | |
| 28S_1057 | 0.134 | 0.123 | 0.079 | | 0.260 | |
| 28S_1116 | 0.183 | 0.161 | 0.019 | | 0.199 | |
| 28S_1167 | 0.547 | 0.530 | 0.242 | 0.337 | |  |
| 28S_1202 | 0.249 | 0.252 | 0.779 | 0.814 | |  |
| 28S_1214 | 0.352 | 0.339 | 0.326 | 0.441 | |  |
| 28S_1303 | 0.201 | 0.194 | 0.544 | 0.610 | |  |
| 28S_1307 | 0.587 | 0.570 | 0.210 | 0.312 | |  |
| 28S_1358 | 0.552 | 0.534 | 0.459 | 0.541 | |  |
| 28S_1384 | 0.259 | 0.243 | 0.426 | 0.541 | |  |
| 28S_1413 | 0.523 | 0.515 | 0.771 | 0.814 | |  |
| 28S_1423 | 0.092 | 0.086 | 0.576 | 0.631 | |  |
| 28S_1568 | 0.632 | 0.611 | 0.059 | 0.209 | |  |
| 28S_1710 | 0.493 | 0.482 | 0.481 | 0.553 | |  |
| 28S_2154 | 0.757 | 0.744 | 0.118 | 0.263 | |  |
| 28S_2656 | 0.068 | 0.065 | 0.442 | 0.541 | |  |
| 28S_2761 | 0.311 | 0.305 | 0.454 | 0.541 | |  |
| 28S_3048 | 0.561 | 0.542 | 0.108 | 0.263 | |  |
| 28S_3538 | 0.583 | 0.585 | 0.916 | 0.916 | |  |
| 28S_3798 | 0.432 | 0.414 | 0.107 | 0.263 | |  |
| 28S_3852 | 0.446 | 0.428 | 0.119 | 0.263 | |  |
| 28S_3915 | 0.711 | 0.701 | 0.154 | 0.272 | |  |
| 28S_3932 | 0.560 | 0.539 | **0.015** | 0.199 | |  |
| 28S_3944 | 0.633 | 0.623 | 0.223 | 0.321 | |  |
| 28S_4042 | 0.681 | 0.660 | **0.014** | 0.199 | |  |

**Table S5** Mean methylation percentage at each locus, raw and Bonferroni–Holm corrected *p*-values from independent samples *t*-test between sexes in the 51 month cohort. Significant *p*-values (< 0.05) in bold and underlined.

| locus | Mean female methylation | Mean male methylation | raw p-value | BH corrected p-value |
| --- | --- | --- | --- | --- |
| 18S_231 | 0.868 | 0.872 | 0.347 | 0.469 |
| 18S_235 | 0.751 | 0.768 | 0.084 | 0.439 |
| 18S_242 | 0.891 | 0.896 | 0.169 | 0.439 |
| 18S_247 | 0.636 | 0.652 | 0.114 | 0.439 |
| 18S_253 | 0.761 | 0.775 | 0.061 | 0.439 |
| 18S_318 | 0.257 | 0.276 | 0.183 | 0.439 |
| 18S_325 | 0.310 | 0.330 | 0.176 | 0.439 |
| 18S_340 | 0.049 | 0.053 | 0.401 | 0.499 |
| 18S_631 | 0.588 | 0.613 | 0.085 | 0.439 |
| 18S_904 | 0.284 | 0.293 | 0.381 | 0.493 |
| 18S_914 | 0.329 | 0.342 | 0.196 | 0.439 |
| 18S_1026 | 0.362 | 0.391 | **0.007** | 0.322 |
| 18S_1304 | 0.196 | 0.202 | 0.543 | 0.609 |
| 18S_1595 | 0.738 | 0.751 | 0.098 | 0.439 |
| 18S_1667 | 0.543 | 0.555 | 0.265 | 0.439 |
| ITS1_1793 | 0.841 | 0.840 | 0.805 | 0.805 |
| ITS1_1874 | 0.233 | 0.243 | 0.386 | 0.493 |
| ITS2_249 | 0.187 | 0.202 | 0.059 | 0.439 |
| ITS2_275 | 0.207 | 0.220 | 0.136 | 0.439 |
| 28S_969 | 0.648 | 0.660 | 0.219 | 0.439 |
| 28S_992 | 0.161 | 0.169 | 0.232 | 0.439 |
| 28S_1029 | 0.279 | 0.294 | 0.272 | 0.439 |
| 28S_1057 | 0.127 | 0.137 | 0.157 | 0.439 |
| 28S_1116 | 0.147 | 0.162 | **0.044** | 0.439 |
| 28S_1167 | 0.526 | 0.541 | 0.252 | 0.439 |
| 28S_1202 | 0.220 | 0.232 | 0.079 | 0.439 |
| 28S_1214 | 0.284 | 0.300 | 0.269 | 0.439 |
| 28S_1303 | 0.185 | 0.195 | 0.277 | 0.439 |
| 28S_1307 | 0.555 | 0.563 | 0.326 | 0.460 |
| 28S_1358 | 0.532 | 0.547 | 0.288 | 0.442 |
| 28S_1384 | 0.228 | 0.237 | 0.159 | 0.439 |
| 28S_1413 | 0.506 | 0.514 | 0.532 | 0.609 |
| 28S_1423 | 0.095 | 0.102 | 0.319 | 0.460 |
| 28S_1568 | 0.625 | 0.641 | 0.121 | 0.439 |
| 28S_1710 | 0.489 | 0.503 | 0.163 | 0.439 |
| 28S_2154 | 0.665 | 0.680 | 0.201 | 0.439 |
| 28S_2656 | 0.223 | 0.227 | 0.702 | 0.745 |
| 28S_2761 | 0.321 | 0.325 | 0.643 | 0.704 |
| 28S_3048 | 0.574 | 0.581 | 0.718 | 0.745 |
| 28S_3538 | 0.610 | 0.625 | 0.274 | 0.439 |
| 28S_3798 | 0.421 | 0.438 | 0.251 | 0.439 |
| 28S_3852 | 0.431 | 0.451 | 0.128 | 0.439 |
| 28S_3915 | 0.732 | 0.740 | 0.330 | 0.460 |
| 28S_3932 | 0.548 | 0.553 | 0.729 | 0.745 |
| 28S_3944 | 0.652 | 0.660 | 0.487 | 0.574 |
| 28S_4042 | 0.656 | 0.663 | 0.412 | 0.499 |

**
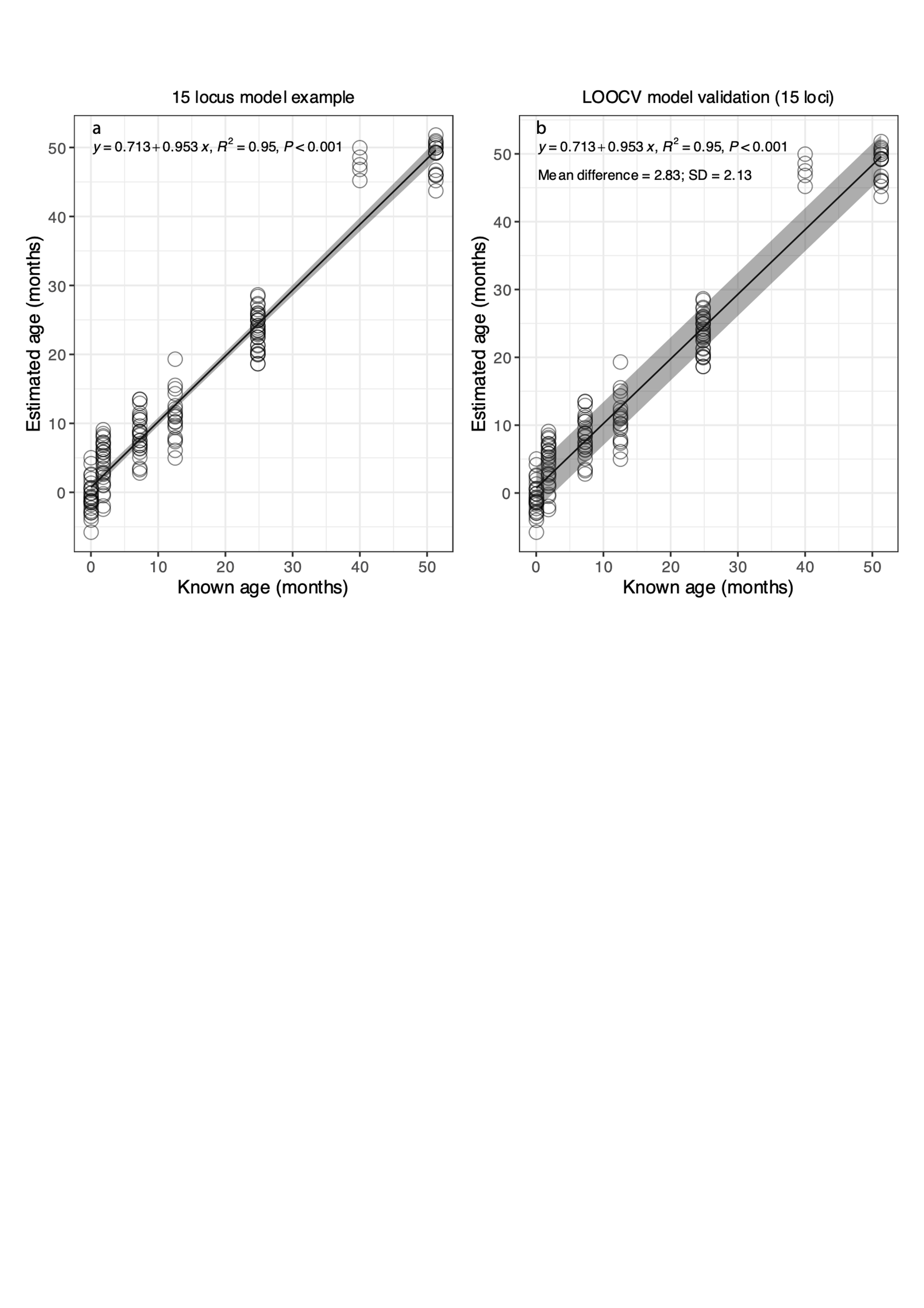
**

**Figure S5a** Multiple linear regression for estimated age based on percentage methylation at 15 CpG loci in European lobsters (*n* = 155). **Figure S5b** the precision of the model as determined using a leave-one-out cross-validation analysis (LOOCV). Grey regions represent the 95% confidence intervals for the regression line in plot a and represents the mean qnorm(0.975)*sd/sqrt(n) of the difference between known and predicted age in plot b

**
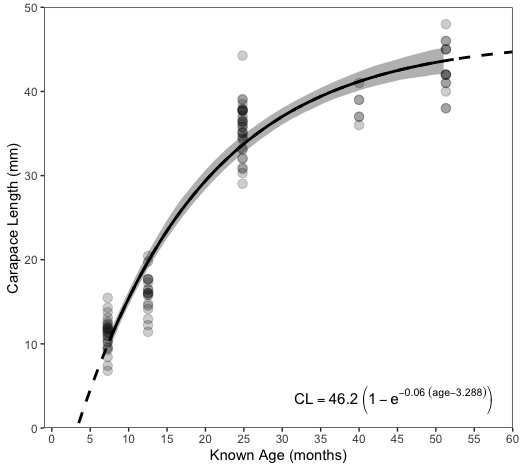
**

**Figure S6** Relationship between (estimated) age and carapace length in known-age individuals. Black line is the von Bertalanffy Growth curve and the grey areas are the bootstrapped 95% confidence intervals. Equation is the best-fit von Bertalanffy Growth Model for Carapace Length (CL).


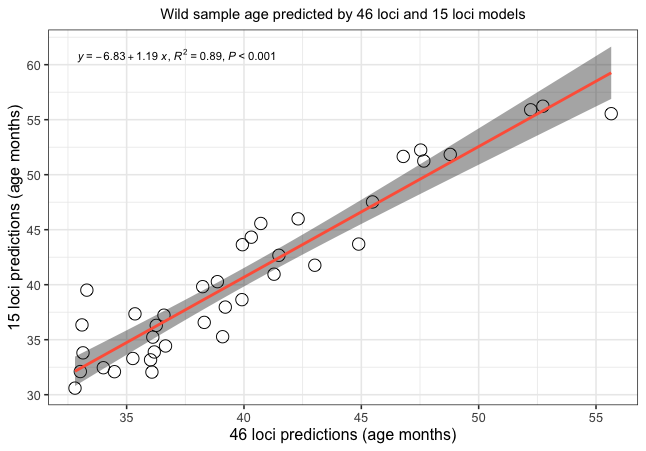


**Figure S7** Predictions of wild age based on the 46 loci Elastic Net model and the 15 loci model. Grey areas represent the 95% confidence intervals.

**Table S6** Estimated methylation saturation ages for the 46 loci included in the ageing model.

| Locus | intercept | coeff | saturation (months) | saturation (years) |
| --- | --- | --- | --- | --- |
| 18S_231 | 0.796 | 0.001 | 154.095 | 12.841 |
| 18S_235 | 0.676 | 0.002 | 205.744 | 17.145 |
| 18S_242 | 0.834 | 0.001 | 156.632 | 13.053 |
| 18S_247 | 0.564 | 0.002 | 253.128 | 21.094 |
| 18S_253 | 0.696 | 0.001 | 229.775 | 19.148 |
| 18S_318 | 0.320 | -0.001 | 249.717 | 20.810 |
| 18S_325 | 0.368 | -0.001 | 272.852 | 22.738 |
| 18S_340 | 0.063 | 0.000 | 193.967 | 16.164 |
| 18S_631 | 0.548 | 0.001 | 321.590 | 26.799 |
| 18S_904 | 0.378 | -0.002 | 205.748 | 17.146 |
| 18S_914 | 0.439 | -0.002 | 246.998 | 20.583 |
| 18S_1026 | 0.366 | 0.001 | 946.367 | 78.864 |
| 18S_1304 | 0.269 | -0.002 | 146.706 | 12.226 |
| 18S_1595 | 0.703 | 0.001 | 368.910 | 30.743 |
| 18S_1667 | 0.583 | -0.001 | 695.285 | 57.940 |
| ITS1_1793 | 0.811 | 0.000 | 458.710 | 38.226 |
| ITS1_1874 | 0.268 | -0.001 | 231.353 | 19.279 |
| ITS2_249 | 0.227 | -0.001 | 225.552 | 18.796 |
| ITS2_275 | 0.251 | -0.001 | 217.544 | 18.129 |
| 28S_969 | 0.642 | 0.000 | 91168.806 | 7597.4 |
| 28S_992 | 0.134 | 0.001 | 1360.607 | 113.384 |
| 28S_1029 | 0.343 | -0.002 | 221.028 | 18.419 |
| 28S_1057 | 0.169 | -0.001 | 166.776 | 13.898 |
| 28S_1116 | 0.144 | 0.001 | 1695.411 | 141.284 |
| 28S_1167 | 0.568 | -0.001 | 624.220 | 52.018 |
| 28S_1202 | 0.262 | -0.001 | 378.531 | 31.544 |
| 28S_1214 | 0.419 | -0.003 | 151.344 | 12.612 |
| 28S_1303 | 0.234 | -0.001 | 203.564 | 16.964 |
| 28S_1307 | 0.585 | -0.001 | 1107.432 | 92.286 |
| 28S_1358 | 0.480 | 0.001 | 360.518 | 30.043 |
| 28S_1384 | 0.224 | 0.000 | 1731.128 | 144.261 |
| 28S_1413 | 0.598 | -0.002 | 261.061 | 21.755 |
| 28S_1423 | 0.138 | -0.001 | 115.779 | 9.648 |
| 28S_1568 | 0.547 | 0.002 | 220.608 | 18.384 |
| 28S_1710 | 0.516 | -0.001 | 702.124 | 58.510 |
| 28S_2154 | 0.782 | -0.002 | 393.205 | 32.767 |
| 28S_2656 | 0.053 | 0.003 | 367.280 | 30.607 |
| 28S_2761 | 0.231 | 0.002 | 349.843 | 29.154 |
| 28S_3048 | 0.535 | 0.001 | 840.856 | 70.071 |
| 28S_3538 | 0.552 | 0.001 | 370.775 | 30.898 |
| 28S_3798 | 0.392 | 0.001 | 800.615 | 66.718 |
| 28S_3852 | 0.459 | -0.001 | 716.059 | 59.672 |
| 28S_3915 | 0.645 | 0.002 | 191.260 | 15.938 |
| 28S_3932 | 0.466 | 0.002 | 250.192 | 20.849 |
| 28S_3944 | 0.606 | 0.001 | 529.358 | 44.113 |
| 28S_4042 | 0.672 | 0.000 | 1782.9 | 148.575 |
